# Quality of Life in Caregivers of Children and Adolescents with Autistic Spectrum Disorder: Development and Validation of the Questionnaire

**DOI:** 10.3390/brainsci11070924

**Published:** 2021-07-13

**Authors:** Claudia B. Pratesi, Alessandra Baeza Garcia, Riccardo Pratesi, Lenora Gandolfi, Mariana Hecht, Eduardo Yoshio Nakano, Renata Puppin Zandonadi

**Affiliations:** 1 Interdisciplinary Laboratory of Biosciences and Celiac Disease Research Center, School of Medicine, University of Brasilia, Brasilia 70910-900, DF, Brazil; alessandrambaeza@gmail.com (A.B.G.); pratesiunb@gmail.com (R.P.); lenoragandolfi1@gmail.com (L.G.); marianahecht@gmail.com (M.H.); 2 Department of Statistics, University of Brasilia, Brasilia 70910-900, DF, Brazil; eynakano@gmail.com; 3 Department of Nutrition, Faculty of Health Sciences, Campus Darcy Ribeiro, University of Brasilia (UnB), Asa Norte, Brasilia 70910-900, DF, Brazil; renatapz@unb.br

**Keywords:** quality of life, autistic spectrum disorder, parents, caregivers

## Abstract

Studies have shown that children and adolescents with autism and their relatives present a high level of stress and more family problems, impacting parents’ and caregivers’ quality of life (QoL). Despite studies on this subject, there is no specific questionnaire to evaluate QoL in parents or caregivers of children and adolescents with an autistic spectrum disorder (ASD) in Brazil. Therefore, this study’s primary purpose was to develop and validate a specific questionnaire to evaluate QoL in these individuals. The study was performed using the following steps: development of the ASD Parent/caregiver QoL questionnaire (autistic spectrum disorder parent/caregiver quality of life—ASDPC-QoL), subjective evaluation, validation of the questionnaire by the Delphi method, assessment of internal consistency, responsiveness, and reliability of the ASLPC-QoL, and administration of the questionnaire to 881 Brazilian ASD caregivers or parents. ASDPC-QoL comprises 28 questions divided into four domains (social, concerns, physical and mental health) with good psychometric properties (reproducibility, reliability, internal consistency, responsiveness, and validity). Our data showed that worries and physical health were the domains with the lowest scores in ASDPCA-QoL. ASDPCA-QoL did not differ among gender and age of child considering the total and all domains. Older participants (≥41 y/o) presented the best scores for social and worries domains but did not differ in other domains and the total. Parents or caregivers of ASD children diagnosed for more than three years have better mental and physical health domains than those recently diagnosed (up to 1 year) but did not differ in the total and other domains. Individuals with a partner and with the highest educational level present the best score for the social domain. Employed individuals showed better scores than unemployed ones for all domains and the total, except for worries, which did not differ. It also occurred comparing the individuals that do not use antidepressants and the ones that use them. Assessing and better understanding the QoL of caregivers is highly relevant. By understanding the social, worries, physical, and emotional health domains of caregivers, it is possible to track harmful aspects, prevent and treat pathologies, in addition to assisting in the implementation of effective public policies.

## 1. Introduction

First described by Leo Kanner in 1943 [1], Autistic Spectrum Disorder (ASD) is described as a global early-onset neurodevelopmental disorder characterized by const communication and social interaction delays in multiple contexts, and restricted and repetitive behavior patterns, interests, or activities. According to epidemiological data from the Centers for Disease Control and Prevention (CDC), the prevalence of ASD is 1 case for every 63 live births [2]. Symptoms are present early in the developmental period, causing significant impairment in school functioning, professional, academic, social, and other essential areas of life [3].

Due to the characteristics of ASD, an autistic child can be a source of stress for parents because they can cause an overload, especially of an emotional nature. The cognitive deficit of these children proved to be more detrimental to their parents than the severity of their symptoms, as they are concerned about their children’s future when they can no longer support them. Some studies show that children and adolescents with autism and their relatives present a high level of stress and more family problems, impacting their quality of life (QoL) [4,5,6]. Thus, it is necessary to evaluate the QoL of their caregivers [7,8,9,10,11,12,13]. 

QoL is a multidimensional concept described as an “individual’s perception of their life in the cultural context and value systems in which they live and their goals, expectations, standards, and concerns” [14]. The patient must be carefully evaluated to avoid confusion between the absence of symptoms versus being fully inserted into society. It is a multidimensional view of medical disorders and a global view of the treatment and outcomes. When measuring QoL, all aspects must be considered, such as treatment, the presence or not of symptoms, and how much damage the body suffers. QoL encompasses physical health, psychological health, level of independence related to mobility, daily activities, drug dependence, medical care, workability, social relations, and the environment in the workplace in which the individual is inserted [14].

QoL assessment has received relevant attention both in clinical practice and research [15] given the need to evaluate other aspects of patients’ lives than the mere identification of symptoms. Generic instruments are used in non-specific populations, evaluating characteristics of QoL. They are used in healthy individuals and can measure the impact of a particular disease on a subject’s life without analyzing specific symptoms. A clear advantage is that generic instruments allow groups of individuals affected by different diseases and conditions to be compared to the general population. As a disadvantage, they may not detect slight changes in QoL due to the specificities of some diseases, as they do not contemplate characteristic symptoms of a particular disease [16]. Sickness Impact Profile (SIP), Nottingham Health Profile (NHP), Medical Outcomes Study, and 36-Item Short-Form Health Survey (SF-36) are examples of generic questionnaires that are designed to reflect the impact of a disease on the lives of patients [17].

Specific instruments are designed to fill the gap left by generic questionnaires. These instruments assess the QoL of a particular disease, so they are more sensitive to identifying and measuring slight QoL differences for a specific population. These instruments focus on the most relevant domains of a particular disease and the characteristics of the patients in which the condition is most prevalent [16]. Such tools can also individually assess specific aspects of QoL, providing greater ability to detect improvement or worsening of the assessed element. Its main feature is its potential to be sensitive to changes in the respondent’s QoL, i.e., its ability to see changes after an intervention. They may be specific for a particular function (physical capacity, sleep, sexual function), in a specific population (elderly, young), for a particular change (pain). Functional Class, Activities of Daily Living (ADL) Instruments, Functional Status Index (FSI), and the Stanford Health Assessment Questionnaire (HAQ) are examples of specific questionnaires designed to assess patient QoL.

Some instruments were used to assess QoL in ASD children and adolescents and their caregivers. Most studies used generic instruments such as HRQoL [5,18,19,20,21]; the U.S. National Survey of Children’s Health (NSCH), which contains some items related to QoL [22]; the Pediatric QoL (PedsQL) Inventory [11]; and WHOQOL-BREF [12,22,23,24]. However, to our knowledge, there are no questionnaires specifically designed to evaluate QoL in parents or caregivers of ASD children and adolescents in Brazil. Therefore, this study aimed to develop and validate a questionnaire to assess the QoL of Brazilian parents or caregivers of ASD children and adolescents.

## 2. Materials and Methods

The study was performed in six steps: (i) development of the ASD parent/caregiver QoL questionnaire (ASDPC-QoL), (ii) subjective evaluation, (iii) validation of the questionnaire utilizing the Delphi method, (iv) evaluation of the internal consistency, responsiveness, and reliability of the ASLPC-QoL, (v) administration of the questionnaire to Brazilian ASD caregivers or parents and (vi) statistical analysis. As a result, the Health Sciences Ethics Committee from the University of Brasilia approved the study under protocol numbers 01223018.8.0000.0030 and 01223018.8.3001.5553, and followed the established guidelines of the Declaration of Helsinki.

### 2.1. Development of the Questionnaire

The questionnaire was developed based on an extensive literature review and the researchers’ experience on the matter. In addition, informal interviews with psychologists, psychiatrists, and other health professionals with expertise in the issue were conducted. In addition to studies evaluating the QoL of patients with chronic illnesses, several general QoL questionnaires were used to design the ASDPC-QoL [19,20,21,22]. Topics and items from previous studies were also evaluated. Those thought to be relevant to assessing the QoL of caregivers of children with ASD were chosen and adapted for the initial questionnaire version. Similar to other studies [25,26,27,28,29,30] of ASLPC-QoL, we adopted four domains: social, worries, physical, and mental health.

### 2.2. Subjective Evaluation, Semantic Evaluation, and Content Validation

The Delphi method was used for content validation based on experts’ opinions to obtain a consensus on a subject in situations where new ideas are being developed [31]. SurveyMonkey^®^ was used for the content validation of the ASDPC-QoL. The first page of the ASDPC-QoL explained the questionnaire’s evaluation criteria.

The validation step is a methodological procedure to evaluate questionnaires’ quality, concerning the instrument’s ability to accurately assess what it is proposed to measure. Therefore, the validation of the ASDPC-QoL was performed in two steps. The first step (semantic evaluation and content validation) was conducted by a panel of experts. The committee defines which items should be maintained, revised, or excluded. For the subjective evaluation, 13 experts, including physicians, psychiatrists, and psychologists with known experience treating ASD, were invited to participate. Of those asked, ten agreed to participate. Once they agreed to participate, experts received the information and guidance to evaluate the questions utilizing the Delphi method.

In the first stage of the evaluation, the expert panel was asked to assess the initial 48 questions developed by the authors. Next, the experts were asked to evaluate each question according to content, clarity, and the items’ consistency. They were also asked to suggest changes, delete, or include items they judged relevantly and were encouraged to comment on improving the questionnaire.

Experts were also asked to assess each question using a 5-point Likert scale ranging from (1) “I fully disagree with the item” to (5) “I fully agree with the item”. Additionally, they were asked if questions should be maintained or not. Once feedback was received, responses were analyzed, and items were then modified or deleted according to their suggestions [24]. After the questionnaire was changed, a new version was sent to the experts for a new round. This process was repeated until a consensus (≥80%) between the experts was achieved.

### 2.3. Reliability Analysis

The reliability of the ASDPC-QoL was evaluated using a convenience sample of 11 ASD parents’ or caregivers’ responses that were invited to participate. They answered the ASDPC-QoL, and one week later, they were asked to answer the ASDPCA-QoL again (participants were not aware that they would have to answer the questionnaire at different moments). The questionnaire’s reliability (using test–retest) was verified by the Intraclass Correlation Coefficient (ICC).

A one-week interval was chosen to minimize possible confounding variables that could affect the results. In the test–retest reliability, the gap between the two responses may vary from a few hours up to several years. As the interval lengthens, test–retest reliability declines because the number of opportunities for the participants or situation to change increases over time. Test–retest studies for health-related QoL instruments use varying intervals between test administrations. Still, most investigators choose intervals ranging from 2 days to 2 weeks [32,33], aiming for a reasonable compromise between recollection bias and unwanted changes.

### 2.4. ASDPCA-QoL Administration in Brazil

The final step was to place the *ASDPCA-QoL* questionnaire on the SurveyMonkey^®^ platform and apply it to a convenience sample of Brazilian ASD parents or caregivers from all regions in Brazil. The questionnaire was available online for four months—between March and June of 2019. According to [34], the process of validating a questionnaire requires 20 respondents per item (20:1). Thus, the minimum sample size was 560 participants to validate this questionnaire composed of 28 items. The inclusion criteria were parents or caregivers of children or adolescents (between 12 months and 18 years) in Brazil diagnosed with ASD by a professional. We excluded those that had children under the age of two or over the age of 18. In addition, individuals were excluded from the survey if their children were not diagnosed with ASD by a physician, had less than a year of diagnosis, or adolescents were over 18 years of age or under 12 months.

The survey’s first page showed the consent form containing exclusion and inclusion criteria. Individuals who agreed to participate signed an electronic form of consent and were directed to the first page of the survey. Those who did not consent were taken to a page thanking them for their time.

### 2.5. Statistical Analysis

The internal consistency of the ASDPCA-QoL and its four domains was verified using Cronbach’s Alpha coefficient, and the responsiveness was evaluated by floor and ceiling effects. The ASDPCA-QoL scores were described using mean and standard deviation (SD), median, and range. Comparison of the ASDPCA-QoL scores and their domains was performed by Student’s *t*-test (for variables with two categories) and by one-way analysis of variance (ANOVA) followed by Tukey’s post hoc test (for variables with three or more categories). The tests were performed considering bilateral hypotheses and a significance level of 5%. The analyses were performed using SPSS (Statistical Package for Social Sciences) version 22.

## 3. Results

### Questionnaire Development, Semantic Evaluation, and Content Validation

**Figure 1:**
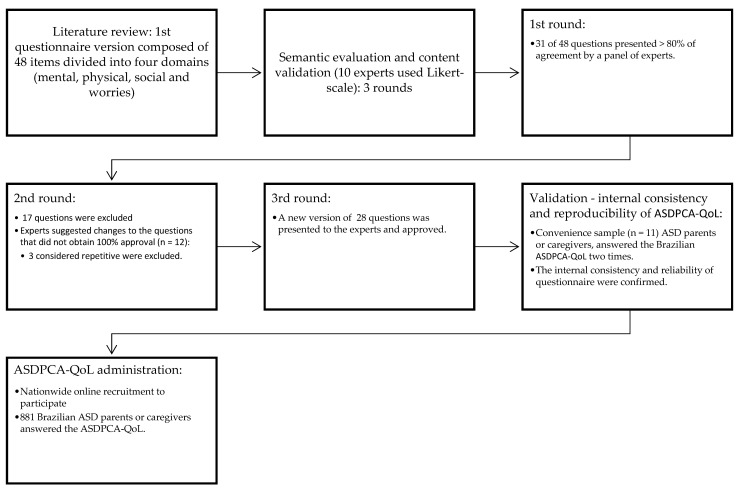
Flowchart of questionnaire development, semantic evaluation, content validation processes, and administration of the ASDPCA-QoL.

The first phase of the study was constructing the initial version of the questionnaire and its evaluation by experts. The initial questionnaire was composed of 48 items (divided into four domains: emotion, worries, and social, physical health) based on an extensive literature review and considering the recommendations made by dietitians, pediatricians, and gastroenterologists with experience with ASD (Figure 1).

After the first version of the questionnaire was constructed, fourteen experts were invited to perform the objective evaluation (semantic evaluation and content validation), and ten agreed to participate. In the first round of objective evaluation, 31 of the 48 questions were approved (≥80% approval rate) based on reliability, clarity, and comprehension, and 17 were excluded. From the 31 approved questions, the experts proposed changes to items that did not obtain 100% approval (*n* = 12). These 12 questions were modified and sent for the 2nd round to be reevaluated. Therefore, we received answers from ten experts; 28 questions were approved, and 3 were excluded because they were deemed repetitive (Figure 1). All questions were approved at the end of the third round, and the final version of the questionnaire presented 28 items (Appendix A).

After the experts approved the final version, the second study phase was performed to verify the internal consistency and reliability of ASDPCA-QoL using a convenience sample of 11 ASD parents/caregivers (eight females, three males aged 30–45). A summary of the construction, semantic evaluation, and content validation processes for ASDPCA-QoL is shown in Figure 1. The ASDPC-QoL (Appendix A) was validated in Brazilian-Portuguese to be used in Brazil. Appendix A also presents a free translation to the English language for best comprehension. Still, to be used in other languages/populations, the instrument must be validated in the target language/population.

In the reliability (test–retest), all four factors of the ASDPCA-QoL showed no significant difference (ICC > 0.7) [35,36] in their responses from the same individual (*n* = 11). The ASDPCA-QoL showed good reliability (ICC = 0.899) (Table 1).

As shown in Table 2, ASDPCA-QoL indicated good internal consistency (α ≥ 0.8) [36]. The questionnaire showed good internal consistency (alpha = 0.884). Only the worries subscale showed sub-ideal consistency (alpha < 0.7). Nevertheless, it did not affect the instrument’s consistency. ASDPCA-QoL showed high responsiveness, presenting low floor and ceiling effects (≤2.5%).

A total of 1085 participants accessed the questionnaire through the SurveyMonkey platform. However, questionnaires were excluded if two or more items were left blank in one of the domains or if the respondents (parents or caregivers) had a child older than 18 y/o. Additionally, some families had more than one child with autism. In this case, for the variables “age of the child” and “age of diagnosis”, only the youngest child was considered. Therefore, of the 1085 responses, 204 were excluded, and the final sample was *n* = 881.

The questionnaire is composed of 28 items with answers on a five-point scale. The index value was defined as the sum of the answers to each of these items. Thus, we can assume values between 28 and 140 for the *ASDPCA-QoL* questionnaire. The higher the score, the greater the QoL. This index is subdivided into four domains (social, worries, physical and mental health), with seven items. Each domain can assume values from 7 to 35. The higher the value of the score, the higher the QoL within the domain. Table 2 showed that worries and physical health were the domains with the lowest scores in *ASDPCA-QoL.*

Table 3 shows the results obtained from the questionnaire application. Most of the respondents were female (*n* = 857; 97.3%); married (*n* = 634; 71.9%); aged between 31 and 40 y/o (*n* = 475; 53.9%); with ASD age up to 12 y/o (85.2%); employed (*n* = 552; 62.6%); income up to four minimum wages (*n* = 702; 79.7%); and most of them did not use antidepressants (*n* = 529; 60%).

ASDPCA-QoL did not differ among gender and age of the child in the total or any of the domains. Older participants (≥41 y/o) presented better social and worries domains scores but did not differ in other domains and total. Parents or caregivers of ASD children diagnosed for more than three years have better mental and physical health domains than those recently diagnosed (up to 1 year) but did not differ in the total or other domains. Individuals with the highest educational level present the best score only in the social domain. Individuals with partners presented better scores in the social domain and total but did not differ in the other two domains. Employed individuals showed better scores than unemployed ones for all domains and the total, except for worries, which did not differ. It also occurred when comparing individuals that do not use antidepressants and the ones that use them.

## 4. Discussion

This is the first study on creating and validating an instrument to assess the QoL of caregivers or parents of children and adolescents with ASD in Brazil. This examination is crucial since ASD during childhood can impair parents/caregivers’ health and QoL because they experience higher stress than healthy children or adolescents. Comprehending the factors that influence the QoL of ASD children’s parents and caregivers may help them achieve optimal health, helping them manage their mental and physical burden and the social limitations imposed by ASD on parents or caregivers. Through an extensive literature review and considering the experts’ suggestions, the ASDPCA-QoL was constructed with 28 questions divided into four domains.

After the semantic evaluation, content validation, and reliability analysis, the ASDPCA-QoL was applied to 881 ASD children or adolescents’ parents or caregivers. As with other studies on QoL [27,28,29,37,38] most participants were female (97.2%; *n*, 857) and had better health, and life expectancy [39]. Having a partner is associated with well-being and quality of life, but the ASDPCA-QoL did not differ considering the participation expected, since females tend to be more concerned about their children’s health according to health studies [40,41].

The presence of an ASD child changes the lives of caregivers and their relationship with family members. Symptoms of the disorder trigger high levels of stress in these families. In our study, the ‘worries’ domain showed the lowest mean, 16.79 ± 5.34, confirming this distress. Families’ social relationships with autistic children diminished, and there may even be disruptions in their social ties [42]. Mothers of children with ASD exhibit lower well-being and higher stress levels than mothers of children with Down syndrome, fragile X syndrome, and cerebral palsy [43]. Other studies also show that families of children with ASD have higher levels of family stress compared to families of children with Down syndrome [44,45] and Attention Deficit Hyperactivity Disorder [22]. Stress, anxiety, and depression are higher in parents of children with ASD when compared to parents of children with other disorders, such as Down Syndrome. Therefore, stress seems to be influenced by specific features of autism and not just by developmental delays [46]. In our sample, 40% of participants (*n* = 349) use antidepressants, potentially related to the mentioned distress caused by ASD.

The social, personal, and financial impacts that these families experience make living with autism an arduous, challenging, exhausting, and sometimes painful task. A family with an autistic member goes through unique experiences, becoming vulnerable to social integration, predisposed to stress and family dysfunction [12,37,47]. It is not uncommon among ASD parents to give up their jobs to take care of their children [48,49]. In our study, almost 40% of participants were unemployed (*n* = 329), confirming this tendency. However, nearly 80% of the participants (*n* = 702) were in the lowest wage level (up to 4 minimum wages (the conversion rate during data collection equated one minimum wage to about 260 dollars)).

The family nucleus suffers immediate disruptions when their routine and activities are interrupted, and the emotional climate in which they live is transformed. The family is united around the child’s difficulties, and this mobilization is crucial at the beginning of the adaptation. The challenges presented by children often make it impossible for families to maintain social norms and values and, consequently, maintain a healthy social life [48]. In our study, a more extended diagnosis indicated better mental and physical QoL domains and total scores.

The way the family deals with the disorder will be influenced by acceptance, and how individuals deal with the daily challenges they face [46]. Thus, the caregiver is a fundamental character for the prognosis of children affected by the disorder. They spend most of their time around the patient, care for their full-time basic needs (hygiene, food, locomotion), and provide emotional and affective support. Caregivers must renounce such things as work, leisure, study, and even personal projects for comprehensive child and adolescent care [50], potentially affecting the social domain. In our research, the social domain was affected by marital status, educational level, employment, income, and use (or not) of antidepressants. Higher social scores were attributed to individuals with higher education and income levels, being married, being employed, and not using antidepressants. Marriage is associated with life happiness, better health, and life expectancy [39]. Having a partner is associated with well-being and quality of life, and the highest financial status and education [39,47,48,49,50,51], which could explain our results that individuals with a partner showed better scores for the social domain and general QoL.

Family members can perceive the care demanded by a child with ASD and changes in family, social and professional routine as a stressful event. Such care has conditions that cause physical and mental overload, altering the perception of QoL by this family [52,53].

Depressive symptoms and higher anxiety levels were found in mothers of children diagnosed with high-functioning autism than mothers of children with typical development [54]. These depressive symptoms could explain the high prevalence of antidepressant use in our sample (Table 3). Additionally, except for the ‘worries’ domain, QoL was worse among individuals using antidepressants.

The quality of the services provided, such as support networks, the availability of financial resources, and the severity of symptoms, influences the caregiver’s QoL [55,56]. However, further studies are necessary to evaluate this in our sample.

This study identified some limitations of how the survey was disseminated (by email and social media) and sampled (convenience sample). However, if we used random sampling, it would not be possible to achieve a large sample, which is a strength of our study. Our sample rate of male participants was low, not allowing our findings to be generalized to other males. Additional studies must consider the inclusion of males to provide a broader comprehension of the ASD child’s parents/caregivers’ QoL to increase generalization.

This search also has essential strengths since ASDPCA-QoL allowed us to assess the impact of child ASD on parents using a specific instrument on a large sample. In addition, this study provides essential data into the potential mechanisms by which caring for a child with ASD burdens parents and caregivers’ QoL. This knowledge may support the development of effective interventions to help ASD parents and caregivers experience excessive burden and stress related to their ASD child, preventing QoL outcomes [14,22,53].

## 5. Conclusions

The main objective of this study was the construction and validation of a specific instrument to assess the QoL of caregivers or parents of children and adolescents. This ASD questionnaire has 28 questions divided into four domains (social, concerns, physical and mental health) with good psychometric properties (reliability, internal consistency, responsiveness, and validity). The large sample involved (881 participants) is significant compared to similar studies.

Assessing caregivers’ QoL is highly relevant because by better understanding physical and emotional health, social and worries issues, it is possible to track harmful aspects, prevent and treat pathologies. Additionally, assessment may assist in better and more effective public policies, resulting in better QoL for this population and, consequently, for those with ASD. Therefore, validating a specific QoL questionnaire for caregivers of children and adolescents with ASD becomes a tool of great relevance, mainly due to the specificities of the disorder.

## Figures and Tables

**Table 1:** Reliability of the instrument and factors of the ASDPCA-QoL (*n* = 11).

	Mean (S.D.) T1	Mean (S.D.) T2	I.C.C.
Mental Health	22.18 (5.23)	22.45 (6.06)	0.927
Physical Health	21.55 (3.47)	22.09 (4.01)	0.811
Social Aspects	21.36 (4.65)	20.91 (6.09)	0.889
Worries	17.64 (5.41)	17.91 (6.49)	0.754
Total Score	82.73 (16.50)	83.3 (20.81)	0.899

**Table 2:** Description, responsiveness, and internal consistency of the instrument and factors of ASDPCA-QoL (*n* = 881).

	Average (Standard Deviation)	Median	Range	Floor Effect (%)	Ceiling Effect (%)	Internal Consistency (Cronbach’s Alpha)
Mental Health	21.80 (5.61)	22 (18–26)	8–35	0%	0.9%	0.758
Physical Health	19.96 (5.23)	20 (16–23)	7–35	0.6%	0.2%	0.732
Social Aspects	20.78 (5.78)	21 (17–21)	7–35	0.3%	0.7%	0.763
Worries	16.79 (5.34)	16 (13–20)	7–33	2.5%	0%	0.670
Total Score	64.82 (14.49)	64 (55–75)	31–106	0%	0%	0.884

**Table 3:** Mean (M), standard deviation (S.D.), and *p*-value of comparison of ASDPCA-QoL questionnaire scores (and their domains) according to study variables (*n* = 881).

	Mental Health		Physical Health		Social Aspects		Worries			Total
	Mean (S.D.)	*p*	Mean (S.D.)	*p*	Mean (S.D.)	*p*	Mean (S.D.)	*p*	Mean (S.D.)	*p*
Gender *										
Female (*n* = 857)	21.78 (5.65) ^a^	0.231	19.93 (5.22) ^a^	0.221	20.73 (5.79) ^a^	0.129	16.74 (5.36) ^a^	0.113	64.70 (15.53) ^a^	0.152
Male (*n* = 24)	22.83 (4.11) ^a^		21.25 (5.27) ^a^		22.54 (5.34) ^a^		18.50 (4.70) ^a^		69.00 (12.28) ^a^	
Age **										
30 and under (*n* = 187)	22.13 (5.70) ^a^		20.62 (5.19) ^a^		20.75 (5.56) ^a^		16.30 (5.61) ^a^		65.86 (14.49) ^a^	
31–40 (*n* = 475)	21.59 (5.66) ^a^	0.492	19.62 (5.05) ^a^	0.075	20.39 (5.67) ^ab^	0.026	16.51 (4.95) ^a^	0.003	63.80 (14.13) ^a^	0.078
41 and over (*n* = 213)	21.94 (5.47) ^a^		20.14 (5.63) ^a^		21.68 (6.17) ^b^		17.86 (5.83) ^b^		66.15 (15.21) ^a^	
Age of Child **										
5 and less (*n* = 412)	22.09 (5.71) ^a^		20.07 (5.05)^a^		20.69 (5.44) ^a^		16.84 (5.23) ^a^		65.21 (14.03) ^a^	
Between 6 and 11 (*n* = 339)	21.57 (5.53) ^a^	0.346	19.99 (5.28) ^a^	0.561	20.78 (5.98) ^a^	0.837	16.63 (5.39) ^a^	0.753	64.65 (14.95) ^a^	0.661
12 and over (*n* = 125)	21.47 (5.57) ^a^		19.50 (5.71) ^a^		21.04 (6.43) ^a^		17.02 (5.59) ^a^		63.91 (15.01) ^a^	
Time Diagnosis **										
Less than one (*n* = 166)	22.79 (5.48) ^a^		20.84 (5.08) ^a^		21.40 (5.66) ^a^		16.78 (5.50) ^a^		67.41 (13.84) ^a^	
Between 1 and 2.99 (*n* = 360)	21.81 (5.74) ^ab^	0.023	19.84 (5.12) ^ab^	0.039	20.69 (5.68) ^a^	0.265	16.91 (5.25) ^a^	0.792	64.73 (14.35) ^ab^	0.020
≥3 years (*n* = 337)	21.33 (5.53) ^b^		19.60 (5.43) ^b^		20.51 (6.04) ^a^		16.63 (5.36) ^a^		63.55 (14.99) ^b^	
Education **										
Elementary School (*n* = 111)	22.12 (5.35) ^a^		19.86 (5.20) ^a^		20.09 (5.64) ^a^		15.90 (5.04) ^a^		64.35 (12.94) ^a^	
High School (*n* = 357)	22.16 (5.65) ^a^	0.198	20.16 (5.30) ^a^	0.768	20.32 (5.79) ^a^	0.010	16.80 (5.26) ^a^	0.196	64.91 (14.80) ^a^	0.978
Undergraduate (*n* = 272)	21.64 (5.45) ^a^		19.91 (5.01) ^a^		20.96 (5.70) ^ab^		16.86 (5.37) ^a^		64.77 (14.04) ^a^	
Post-Graduate (*n* = 140)	21.04 (5.95) ^a^		19.64 (5.53) ^a^		22.10 (5.87) ^b^		17.36 (5.71) ^a^		65.13 (15.81) ^a^	
Marital status *										
Single (*n* = 245)	21.44 (5.43) ^a^	0.220	19.54 (5.26) ^a^	0.127	20.05 (5.83) ^a^	0.020	16.52 (5.43) ^a^	0.357	63.27 (14.32) ^a^	0.044
With partner (*n* = 634)	21.96 (5.67) ^a^		20.14 (5.21) ^a^		21.06 (5.74) ^b^		16.89 (5.31) ^a^		65.46 (14.51) ^b^	
Employed *										
No (*n* = 328)	21.30 (5.68) ^a^	0.042	19.06 (5.22) ^a^	<0.001	19.57 (5.79) ^a^	<0.001	16.38 (5.05) ^a^	0.081	62.23 (14.42) ^a^	<0.001
Yes (*n* = 552)	22.10 (5.56) ^b^		20.49 (5.16) ^b^		21.48 (5.67) ^b^		17.03 (5.50) ^a^		66.34 (14.33) ^b^	
Income **										
Up to 2 MW (*n* = 486)	21.84 (5.73) ^a^		19.91 (5.28) ^a^		20.32 (5.90) ^a^		16.30 (5.27) ^a^		64.29 (14.79) ^a^	
2.01 a 4 MW (*n* = 216)	21.90 (5.11) ^a^	0.201	20.11 (4.80) ^a^	0.464	20.92 (5.38) ^ab^	0.010	17.04 (5.29) ^ab^	0.010	65.25 (13.11) ^a^	0.215
4.01 a 10 MW (*n* = 123)	22.17 (5.75) ^a^		20.40 (5.65) ^a^		22.07 (5.77) ^b^		18.05 (5.27) ^b^		67.11 (15.26) ^a^	
More than 10 M.W. (*n* = 42)	20.07 (5.85) ^a^		18.98 (4.99) ^a^		22.05 (5.47) ^b^		16.98 (6.13) ^ab^		63.12 (14.66) ^a^	
Antidepressant *										
No (*n* = 529)	22.74 (5.65) ^a^	<0.001	20.85 (5.00) ^a^	<0.001	21.45 (5.68) ^a^	<0.001	17.05 (5.43) ^a^	0.059	67.36 (14.18) ^a^	<0.001
Yes (*n* = 349)	20.37 (5.27) ^b^		18.60 (5.29) ^b^		19.72 (5.80) ^b^		16.36 (5.16) ^a^		60.89 (14.12) ^b^	

Not all answers total *n* = 881 because some individuals did not answer all socioeconomic questions. * Student *t*-test, ** ANOVA with post hoc Tukey. Groups with the same letters do not differ significantly. Categories. Marital Status—With life Partner: married or in a stable relationship; no partner: Divorced, single, widowed.

## Data Availability

The study did not report any data.

## Appendix A. Questionário Sobre Qualidade de Vida de Cuidadores de Crianças e Adolescentes com TEA—Autistic Spectrum Disorder Parent/Caregiver Quality of Life—ASDPC-QoL

**Saúde Mental/Mental Health**
1. Teve dificuldades em relação a sua vida íntima afetiva, nas duas últimas semana? Have you had difficulties in relation to your intimate affective life in the last two weeks?
( ) Sempre/Always
( ) Quase sempre/Almost always
( ) Algumas vezes/Sometimes
( ) Quase nunca/Almost never
( ) Nunca/never
2. Nas duas últimas semanas, envolveu-se em brigas ou discussões com o companheiro (a) por causa de seu filho (a)? In the past two weeks, have you been involved in fights or arguments with your partner over your child?
( ) Sempre
( ) Quase sempre
( ) Algumas vezes
( ) Quase nunca
( ) Nunca
3. Nas duas últimas semanas, pensou na necessidade de ajuda psicológica? In the past two weeks, have you thought about the need for psychological help?
( ) Sempre
( ) Quase sempre
( ) Algumas vezes
( ) Quase nunca
( ) Nunca
4. Durante uma situação de estresse com o seu filho (a) por ex.: agitação psicomotora, agressividade, birra, você consegue manter-se calma (o) para resolver a situação? During a stressful situation with your child, e.g., psychomotor agitation, aggression, tantrum, can you keep calm to resolve the situation?
( ) Nunca mantenho a calma
( ) Quase nunca mantenho a calma
( ) Algumas vezes mantenho a calma
( ) Quase sempre mantenho a calma
( ) Sempre mantenho a calma
5. Houve mudança na relação com seu companheiro (a) após a descoberta do diagnóstico? Was there a change in the relationship with your partner after discovering the diagnosis?
( ) Sempre
( ) Quase sempre
( ) Algumas vezes
( ) Quase nunca
( ) Nunca
6. Nas duas últimas semanas, sentiu-se triste ou deprimida (o) ou chorosa (o)? In the past two weeks, have you felt sad or depressed or tearful?
( ) Sempre
( ) Quase sempre
( ) Algumas vezes
( ) Quase nunca
( ) Nunca
7. Teve pensamentos sobre tirar a própria vida após a descoberta do diagnóstico de seu filho (a)? Have you had thoughts about taking your own life after your child’s diagnosis?
( ) Sempre
( ) Quase sempre
( ) Algumas vezes
( ) Quase nunca
( ) Nunca
**Saúde Física/Physical Health**
8. O consumo de álcool, tabaco, ou outras drogas aumentou após a descoberta do diagnóstico? Did the consumption of alcohol, tobacco, or other drugs increase after the diagnosis was discovered?
( ) Aumentou muito
( ) Aumentou moderadamente
( ) Aumentou mais ou menos
( ) Aumentou pouco
( ) Não aumentou
9. Teve dificuldades para dormir nas duas últimas semanas? Have you had difficulty sleeping in the past two weeks?
( ) Sempre
( ) Quase sempre
( ) Algumas vezes
( ) Quase nunca
( ) Nunca
10. O fato de ter de cuidar de seu filho (a) a (o) impossibilitou ou a (o) deixou indisposta (o) para a prática de atividades físicas? Did the fact of having to take care of your child make you unable or unwilling to practice physical activities?
( ) Sempre
( ) Quase sempre
( ) Algumas vezes
( ) Quase nunca
( ) Nunca
11. Com que frequência você cuida da sua saúde? How often do you take care of your health?
( ) Nunca
( ) Quase nunca
( ) Algumas vezes
( ) Quase sempre
( ) Sempre
12. Com que frequência, nas duas últimas semanas, sentiu-se cansada (o) ao realizar suas atividades diárias? How often, in the past two weeks, did you feel tired while performing your daily activities?
( ) Sempre
( ) Quase sempre
( ) Algumas vezes
( ) Quase nunca
( ) Nunca
13. Com que frequência, nas duas últimas semanas sentiu dores físicas? How often in the past two weeks did you experience physical pain?
( ) Sempre
( ) Quase sempre
( ) Algumas vezes
( ) Quase nunca
( ) Nunca
14. Após a descoberta do transtorno de seu filho (a) você desenvolveu/piorou alguma doença crônica como por exemplo: diabetes, hipertensão arterial ou doenças autoimunes? After discovering your child’s disorder, did you develop/worse any chronic disease such as diabetes, high blood pressure or autoimmune diseases?
( ) Sempre
( ) Quase sempre
( ) Algumas vezes
( ) Quase nunca
( ) Nunca
**Aspectos Sociais/Social Aspects**
15. Com que frequência você evitou sair em lugares públicos com seu filho (a)? How often have you avoided going out in public places with your child?
( ) Sempre
( ) Quase sempre
( ) Algumas vezes
( ) Quase nunca
( ) Nunca
16. Nas duas últimas semanas, com que frequência recebeu apoio de familiares ou amigos quando necessitou? Over the last few weeks, how often have you received support from family or friends when you need it?
( ) Nunca
( ) Quase nunca
( ) Algumas vezes
( ) Quase sempre
( ) Sempre
17. Nas ultimas duas semanas, com que frequência deixou de frequentar festas ou eventos sociais com a sua família ou com seus amigos? In the past two weeks, how often did you miss going to parties or social events with your family or friends?
( ) Sempre
( ) Quase sempre
( ) Algumas vezes
( ) Quase nunca
( ) Nunca
18. Nas duas ultimas semanas, teve alguém para compartilhar suas dificuldades? In the past two weeks, have you had someone to share your struggles with?
( ) Nunca
( ) Quase nunca
( ) Algumas vezes
( ) Quase sempre
( ) Sempre
19. Nas duas últimas semanas, com que frequência você teve algum momento de lazer com amigas (os)? In the past two weeks, how often have you had any leisure time with friends?
( ) Nunca
( ) Quase nunca
( ) Algumas vezes
( ) Quase sempre
( ) Sempre
20. Nas duas ultimas semanas, ao sair com seu filho (a) sentiu-se envergonhada (o) ou discriminada (o) por algum comportamento atípico apresentado por seu filho (a)? In the last two weeks, when you went out with your child, did you feel ashamed or discriminated against for any atypical behavior presented by your child?
( ) Sempre
( ) Quase sempre
( ) Algumas vezes
( ) Quase nunca
( ) Nunca
21. Evitou sair, nas duas ultimas semanas, por conta de dificuldades relacionadas à alimentação de seu filho? Have you avoided going out in the last two weeks due to difficulties related to feeding your child?
( ) Sempre
( ) Quase sempre
( ) Algumas vezes
( ) Quase nunca
( ) Nunca
**Preocupações/Preocupations**
22. Nas duas últimas semanas, você se preocupou com a possibilidade de seu filho (a) sofrer bullying ou algum tipo de discriminação? In the past two weeks, have you been concerned about your child being bullied or discriminated against?
( ) Sempre
( ) Quase sempre
( ) Algumas vezes
( ) Quase nunca
( ) Nunca
23. Nas duas últimas semanas, teve preocupações com a possibilidade de ter feito algo que possa ter causado TEA em seu filho (a)? In the past two weeks, you have had concerns about the possibility of having done something that may have caused ASD in your child?
( ) Sempre
( ) Quase sempre
( ) Algumas vezes
( ) Quase nunca
( ) Nunca
24. Teve preocupações, nas ultimas duas semanas, relacionadas ao fato de quem irá cuidar de seu filho (a) se algo acontecer com você? Have you had concerns in the past two weeks about who will take care of your child if something happens to you?
( ) Sempre
( ) Quase sempre
( ) Algumas vezes
( ) Quase nunca
( ) Nunca
25. Nas duas últimas semanas, se preocupou com o fato de se dedicar mais ao (a) filho(a) com o transtorno do que outros membros da família? In the past two weeks, were you concerned about being more dedicated to the child with the disorder than other family members?
( ) Sempre
( ) Quase sempre
( ) Algumas vezes
( ) Quase nunca
( ) Nunca
26. Nas duas ultimas semanas, se preocupou com a possibilidade de ser abandonada (o) pelo companheiro (a) por este não conseguir lidar com o transtorno de seu filho (a)? In the last two weeks, were you worried about the possibility of being abandoned by your partner because he/she cannot deal with your child’s disorder?
( ) Sempre
( ) Quase sempre
( ) Algumas vezes
( ) Quase nunca
( ) Nunca
27. Nas duas ultimas semanas, preocupou-se mais com o bem estar de seu filho (a) do que com o seu? In the last two weeks, have you been more concerned with your child’s well-being than with yours?
( ) Sempre
( ) Quase sempre
( ) Algumas vezes
( ) Quase nunca
( ) Nunca
28. Teve preocupações, nas duas ultimas semanas, com a possibilidade de seu filho (a) ser dependente financeiramente dos pais na vida adulta? Have you had concerns, in the last two weeks, with the possibility of your child being financially dependent on the parents in adulthood?
( ) Sempre
( ) Quase sempre
( ) Algumas vezes
( ) Quase nunca
( ) Nunca
